# A w-ACT model for sarcopenia among community-dwelling older adults based on National Basic Public Health Services: development and validation study

**DOI:** 10.3389/fpubh.2025.1522903

**Published:** 2025-08-26

**Authors:** Huanhuan Huang, Siqi Jiang, Zhiyu Chen, Xinyu Yu, Keke Ren, Qinghua Zhao

**Affiliations:** ^1^Department of Nursing, The First Affiliated Hospital of Chongqing Medical University, Chongqing, China; ^2^Nursing Research Center, The First Affiliated Hospital of Chongqing Medical University, Chongqing, China; ^3^Department of orthopedics, The First Affiliated Hospital of Chongqing Medical University, Chongqing, China

**Keywords:** sarcopenia, older adults, risk, machine learning, community

## Abstract

**Background:**

Sarcopenia leads to substantial health and well-being impairments in older adults, underscoring the need for early detection to facilitate intervention. Despite its importance, community settings face challenges with data accessibility, model interpretability, and predictive accuracy.

**Objective:**

To develop a local, data-driven, machine learning-based predictive model aimed at identifying high-risk sarcopenia populations among community-dwelling older adults.

**Methods:**

The study encompassed 910 participants over 60 years old from the National Basic Public Health Services (NBPHS) program. Sarcopenia was ascertained by the Asian Working Group for Sarcopenia (AWGS) criteria. We leveraged Logistic Regression and seven additional machine learning models for risk prediction, employing the LASSO method for feature selection, employing LASSO regression with 10-fold cross-validation for feature selection. The optimal lambda.1se threshold identified four key predictors forming the w-ACT model (weight, Age, Calf circumference, Triglycerides). A comprehensive set of 10 diagnostic indicators was utilized to assess model performance.

**Results:**

The Random Forest-based w-ACT model demonstrated superior performance, with an AUC of 0.872 (95%CI: 0.793,0.950) (validation set) and MCC of 0.566, 0.841 (95%CI: 0.777,0.904) (test set) and MCC of 0.511. Key predictors included weight, age, calf circumference, and triglycerides. SHAP analysis confirmed clinical interpretability.

**Conclusion:**

The w-ACT model offers a reliable, interpretable tool for community-based sarcopenia screening, leveraging accessible variables to guide preventive care.

## 1 Introduction

Sarcopenia, characterized by the progressive loss of muscle mass, strength, and function, is a prevalent condition among older adults (1). Its prevalence ranges from 10 to 27% in individuals aged over 60 years (2). The impact of sarcopenia on older adults is multifaceted, encompassing various dimensions of health and well-being. Research has demonstrated that sarcopenia is correlated with functional impairment, physical disability, and an elevated susceptibility to adverse health-related outcomes (3). Furthermore, as the largest metabolic organ of the human body, the dysfunction of skeletal muscle is also associated with the increased risk of osteoporosis (4), diabetes metabolism (5), cardiovascular diseases (6), and other chronic diseases, which ultimately leads to the decease of physical activity, and limitations in health-related quality of life (7). Given the detrimental impact of sarcopenia, multiple consensus underscores the significance of early detection, intervention, and management strategies (8, 9).

In recent years, propelled by advancements in machine learning and artificial intelligence, researchers have employed statistical or machine learning models to forecast specific health-related outcomes (10). These models have the capacity to analyze complex healthcare data and provide valuable insights for disease risk, treatment response, or healthcare resource utilization (11). The prediction of sarcopenia has been a subject of extensive research, with various models and tools being developed to identify and predict the risk of sarcopenia in different patient populations. For instance, Shin et al. developed a predictive model for sarcopenia utilizing multiple biomarkers in community-dwelling older adults (12). Similarly, Xu et al. developed a multivariable model based on ultrasound imaging features of the gastrocnemius muscle to identify patients with sarcopenia (13). Several researchers developed and validated of a nomogram for predicting sarcopenia in community-dwelling older adults (14, 15).

While current studies collectively support the feasibility of using physiological, biochemical, and imaging indicators to predict sarcopenia, existing sarcopenia prediction models face critical limitations in community settings. One of the primary limitations is the availability of data. The development of predictive models typically requires a large amount of clinical data. However, compared to hospital settings, there may be difficulties in obtaining older adult patients’ information and test results in community setting. Thus, the reliance on specific modalities such as ultrasound and serum biomarkers for risk stratification may pose limitations in terms of accessibility and standardization (16, 17). Another critical limitation is the transferability of model. Current evidence emphasizes specific population groups, such as cancers patients (18) and chronic patients (19–21), which may hinder the widespread application of community screening of certain prediction models, necessitating the development of cost-effective, universal screening methods. Additionally, the lack of data from China using precise assessments for sarcopenia, such as skeletal muscle area or skeletal muscle mass index, underscores the need for more comprehensive and standardized diagnostic criteria. Thus, this highlights the importance of highly actionable, wide applicability, and well-performance insights in the implementation of prediction models in sarcopenia.

China’s National Basic Public Health Services (NBPHS) program, launched in 2009 by the Chinese government, is a nationwide healthcare initiative designed to enhance population health by delivering essential preventive and primary care services (22). A key component of the program is annual health examinations for adults aged 60 and above, conducted at local community health centers (23). The NBPHS dataset offers several unique advantages. Firstly, unlike hospital-based datasets, NBPHS captures community-dwelling older adults without selection bias (e.g., excluding those with severe comorbidities). Secondly, prior studies relied on niche biomarkers (e.g., serum leptin) or imaging (e.g., CT) that are impractical for community screening. NBPHS data bridge this gap by using accessible, low-cost variables scalable to resource-limited settings. More importantly, the program’s nationwide infrastructure and standardized protocols ensure that any risk models developed from these data can be immediately integrated into existing community health workflows.

In this study, we developed and validated a machine learning model (w-ACT: weight, Age, Calf circumference, Triglycerides) using routinely collected NBPHS variables to predict sarcopenia risk among community-dwelling older adults. Through comparative analysis of eight algorithms, our Random Forest-based model achieved superior performance while maintaining clinical interpretability. The final w-ACT model incorporates four readily accessible predictors—weight, Age, Calf circumference, and Triglycerides—addressing critical gaps in community screening through three key innovations: (1) replacing specialized biomarkers with routine examination metrics, (2) demonstrating cross-regional generalizability through external validation, and (3) providing transparent risk stratification. This approach establishes a practical framework for integrating sarcopenia risk assessment into China’s primary care system while maintaining diagnostic accuracy comparable to resource-intensive methods.

## 2 Materials and methods

### 2.1 Study design

**Figure 1:**
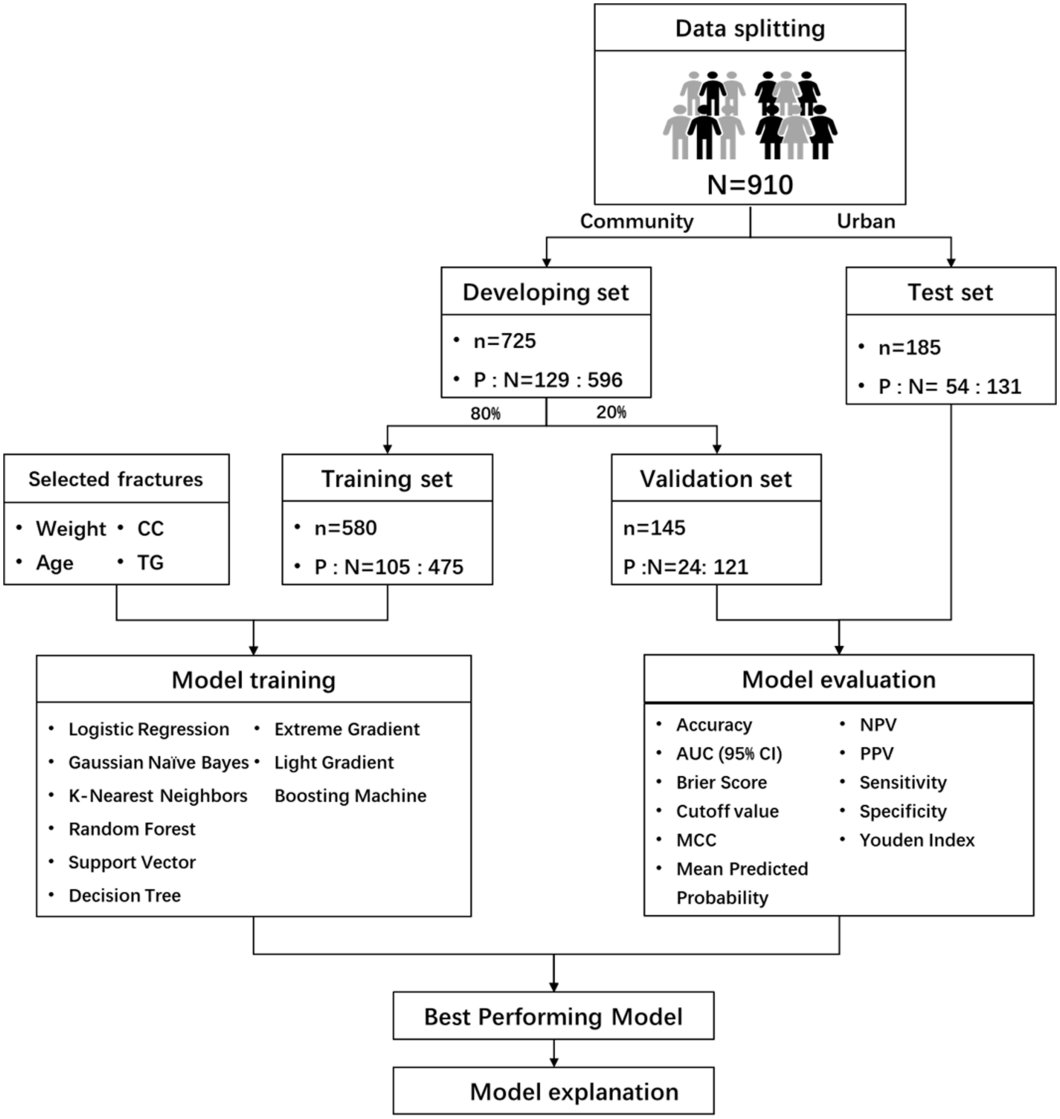
Flowchart summary of study methodology.

This study follows the Declaration of Helsinki and has been approved by the Ethics Committee of Chongqing Medical University (Approval number: 2022-125), written consent was obtained each participant. This study adheres to the Transparent Reporting of a Multivariable Prediction Model for Individual Prognosis or Diagnosis (TRIPOD) reporting guidelines (24). The flowchart of the methodology was shown in Figure 1.

### 2.2 Participants and data sources

#### 2.2.1 Inclusion and exclusion criteria

Inclusion criteria for the study were: participation in the NBPHS program, age over 60 years, and voluntary participation with informed consent. Exclusion criteria include: contraindications for Bioelectrical Impedance Analysis (BIA) such as pacemakers or artificial joints; presence of clinically visible edema or use of diuretics affecting BIA; severe cognitive impairment (Minimum Mental State Examination score ≤ 10); severe disability (Activities of Daily Living scale score ≤ 40); patients in the terminal or acute phase of illness; and patients with muscle and nerve diseases such as genetic muscular dystrophy, mitochondrial diseases, myalgia, and myopathy.

#### 2.2.2 Sample size

To develop a robust prediction model, we followed four-step sample size calculation (25). Considering a binary outcome model, we set an acceptable difference of 0.05 in R^2^ and a margin of error of 0.05 for intercept estimation. With a primary outcome measure proportion of 0.175 from our previous study, 22 predictor variables, an expected shrinkage factor ≤10%, a C-statistic of 0.939 (26), and a Cox & Snell R^2^ of 0.382, we calculated a minimum sample size of 565 participants for model development, equating to 99 events and 4.49 events per predictor.

#### 2.2.3 Outcome variable

Outcome of interest was sarcopenia (0 = non-sarcopenic group, 1 = sarcopenic group) and assessed by Asian Working Group for Sarcopenia (AWGS) in 2019 (1).

### 2.3 Data sources

#### 2.3.1 Setting

Participants were recruited from four community health centers in Chongqing and one rural center from May to October 2023.

#### 2.3.2 Data collection

According to the third edition of the NBPHS guidelines (27), this study includes four categories: general background information, lifestyle and health assessment, physical examination, and auxiliary examinations, totaling 59 predictive indicators.

### 2.4 Data preprocessing and features selecting

For model development, community dataset data were split 8:2 for training and internal validation using the caret package. County data were used for external validation. To handle missing values, multiple imputation was used instead of direct exclusion to prevent bias and loss of statistical power. Features with over 30% missing values were removed (28, 29). Data was normalized using Z-score transformation, and random oversampling addressed class imbalance, referring to previous research (30). The LASSO method with a 10-fold cross-validation was used to select features. No participants were excluded due to missing data; multiple imputation allowed full utilization of all available records while minimizing bias.

### 2.5 Model developing, evaluating and explaining

For model development, we followed guidelines for biomedical machine learning prediction models (31) and used Logistic Regression and seven ML models with the Tidymodels package. Models were validated with internal and external datasets. Discrimination was assessed using 10 indicators. Scores ranged from 1 to 8, with higher scores indicating better performance, except for the Brier Score. The overall model performance score was calculated by summing individual scores. Unlike accuracy or F1-score, Matthews Correlation Coefficient (MCC) accounts for all four confusion matrix categories (TP, TN, FP, FN), making it particularly informative for binary classification with imbalanced classes (32). Thus, MCC was selected as the primary comparison metric. This method has been reported in previous studies (33, 34).

The best-performing model was analyzed using SHAP (SHapley Additive exPlanations) values to quantify and visualize feature importance, including the directionality and magnitude of each predictor’s effect on sarcopenia risk (35), supplemented by the iBreakDown package for perturbation-based validation (36).

### 2.6 Statistical analysis

For continuous variables with normal distribution, mean and SD are reported; for skewed data, mean and IQR are used. Independent *t*-tests compare continuous variables, while chi-square or Fisher’s exact tests compare categorical variables between participants with and without sarcopenia. Analyses were done using R software version 4.0.2, with significance at *p* < 0.05.

## 3 Results

### 3.1 Participants characteristics

A total of 1,000 older adult individuals were initially screened. After excluding 15 participants with incomplete data, 53 with missing diagnostic data, and 22 with BIA contraindications, our final analytical sample comprised 910 participants. The cohort’s mean age was 71.76 ± 5.67 years, with 44.2% being male. Sarcopenia prevalence was 20.1% (183 cases). Complete demographic characteristics stratified by sarcopenia status are presented in Table 1, while detailed demographic and clinical characteristics for each subset provided in Supplementary Table S1 (training), Supplementary Table S2 (validation), and Supplementary Table S3 (test).

**Table 1:** Demographic and clinical characteristics of full participants (*n* = 910).

Variables	Categories	Overall (*n* = 910)	Non-sarcopenia (*n* = 727)	Sarcopenia (*n* = 183)	*p*-value
Gender (%)	Female	508 (55.8)	405 (55.7)	103 (56.3)	0.955
	Male	402 (44.2)	322 (44.3)	80 (43.7)	
Education level (%)	Illiterate	103 (11.3)	76 (10.5)	27 (14.8)	0.029
	Elementary education	140 (15.4)	102 (14.0)	38 (20.8)	
	Junior school education	375 (41.2)	310 (42.6)	65 (35.5)	
	High school education	175 (19.2)	139 (19.1)	36 (19.7)	
	Tertiary education	117 (12.9)	100 (13.8)	17 (9.3)	
Marital status (%)	Married	703 (77.3)	569 (78.3)	134 (73.2)	0.063
	Divorced	17 (1.9)	15 (2.1)	2 (1.1)	
	Widowed	130 (14.3)	93 (12.8)	37 (20.2)	
	Other/ Prefer not to answer	60 (6.6)	50 (6.9)	10 (5.5)	
Employment status (%)	Retired	839 (92.2)	671 (92.3)	168 (91.8)	0.945
	Engaged in farming/work	71 (7.8)	56 (7.7)	15 (8.2)	
Living situation (%)	Living alone	139 (15.3)	113 (15.5)	26 (14.2)	0.031
	Living with spouse	457 (50.2)	373 (51.3)	84 (45.9)	
	Living with children	126 (13.8)	88 (12.1)	38 (20.8)	
	Living with spouse and children	159 (17.5)	127 (17.5)	32 (17.5)	
	Others	29 (3.2)	26 (3.6)	3 (1.6)	
Medical insurance status (%)	Resident medical insurance	135 (14.8)	104 (14.3)	31 (16.9)	0.002
	Employee medical insurance	704 (77.4)	577 (79.4)	127 (69.4)	
	Others	71 (7.8)	46 (6.3)	25 (13.7)	
Overall assessment of your health status (%)	Very poor	81 (8.9)	59 (8.1)	22 (12.0)	0.001
	Relatively poor	354 (38.9)	266 (36.6)	88 (48.1)	
	Relatively good	420 (46.2)	352 (48.4)	68 (37.2)	
	Very good	55 (6.0)	50 (6.9)	5 (2.7)	
Traditional Chinese medicine constitution (%)	Balanced constitution	155 (17.0)	117 (16.1)	38 (20.8)	<0.001
	Dampness-heat constitution	75 (8.2)	51 (7.0)	24 (13.1)	
	Phlegm-dampness constitution	151 (16.6)	120 (16.5)	31 (16.9)	
	Qi-deficiency constitution	243 (26.7)	227 (31.2)	16 (8.7)	
	Yang-deficiency constitution	277 (30.4)	204 (28.1)	73 (39.9)	
	Yin-deficiency constitution	9 (1.0)	8 (1.1)	1 (0.5)	
Average sleep time in the past month (%)	<6 h	349 (38.4)	280 (38.5)	69 (37.7)	0.035
	6 ~ 8 h	408 (44.8)	336 (46.2)	72 (39.3)	
	>8 h	153 (16.8)	111 (15.3)	42 (23.0)	
Average daily step count in the last 3 days (%)	<2000 steps	156 (17.1)	111 (15.3)	45 (24.6)	<0.001
	2000 ~ 4,000 steps	164 (18.0)	119 (16.4)	45 (24.6)	
	4,000 ~ 6,000 steps	193 (21.2)	162 (22.3)	31 (16.9)	
	6,000 ~ 8,000 steps	159 (17.5)	125 (17.2)	34 (18.6)	
	8,000 ~ 10,000 steps	63 (6.9)	56 (7.7)	7 (3.8)	
	>10,000 steps	175 (19.2)	154 (21.2)	21 (11.5)	
Hypertension (%)	No	338 (37.1)	267 (36.7)	71 (38.8)	0.665
	Yes	572 (62.9)	460 (63.3)	112 (61.2)	
Diabetes (%)	No	611 (67.1)	484 (66.6)	127 (69.4)	0.523
	Yes	299 (32.9)	243 (33.4)	56 (30.6)	
Smoking in the past 3 months (%)	No	794 (87.3)	633 (87.1)	161 (88.0)	0.837
	Yes	116 (12.7)	94 (12.9)	22 (12.0)	
Drinking alcohol in the past 3 months (%)	No	805 (88.5)	636 (87.5)	169 (92.3)	0.087
	Yes	105 (11.5)	91 (12.5)	14 (7.7)	
Pain symptoms in the past 3 months (%)	No	856 (94.1)	687 (94.5)	169 (92.3)	0.355
	Yes	54 (5.9)	40 (5.5)	14 (7.7)	
Falls in the past year (%)	No	791 (86.9)	640 (88.0)	151 (82.5)	0.063
	Yes	119 (13.1)	87 (12.0)	32 (17.5)	
Grip strength (Kg, SD)		23.95 (7.96)	25.46 (7.81)	17.98 (5.32)	<0.001
SMI (Kg/m^2^, SD)		6.451 (1.419)	6.680 (1.455)	5.540 (0.750)	<0.001
Age		71.746 (5.685)	71.045 (5.114)	74.530 (6.883)	<0.001
Height (cm)		156.341 (8.514)	157.261 (8.313)	152.686 (8.344)	<0.001
Total weight (Kg)		59.765 (9.593)	61.839 (9.017)	51.525 (7.059)	<0.001
Waist circumference (cm)		84.341 (9.789)	85.492 (9.903)	79.765 (7.816)	<0.001
Calf circumference (cm)		33.953 (3.417)	34.549 (3.183)	31.587 (3.292)	<0.001
BMI (kg/m^2^)		24.431 (3.343)	25.014 (3.247)	22.115 (2.650)	<0.001
Waist-hip ratio (−)		0.885 (0.058)	0.891 (0.057)	0.860 (0.054)	<0.001
White blood cell count (cells/μL)		5.532 (1.441)	5.536 (1.441)	5.516 (1.443)	0.869
Red blood cells (million/μL)		4.821 (0.600)	4.851 (0.596)	4.702 (0.603)	0.003
Red cell distribution width coefficient of variation (−)		13.970 (1.061)	13.927 (1.040)	14.142 (1.129)	0.014
Hematocrit (%)		48.387 (14.848)	48.382 (6.736)	48.408 (30.334)	0.983
Lymphocyte percentage (μm)		30.339 (7.611)	30.536 (7.623)	29.557 (7.534)	0.12
Red cell distribution width standard deviation (μm)		53.035 (6.024)	53.195 (5.763)	52.398 (6.949)	0.11
Lymphocyte count (thousand/μL)		1.642 (0.493)	1.654 (0.495)	1.597 (0.484)	0.166
Mean corpuscular hemoglobin content (pg/cell)		28.925 (2.682)	29.002 (2.615)	28.619 (2.923)	0.084
Neutrophil percentage (%)		60.051 (8.269)	59.902 (8.251)	60.643 (8.339)	0.279
Mean corpuscular hemoglobin concentration (g/L)		291.868 (24.770)	291.292 (24.658)	294.158 (25.146)	0.162
Neutrophil count (thousand/μL)		3.394 (1.317)	3.394 (1.363)	3.392 (1.121)	0.983
Mean corpuscular volume (fL)		99.350 (9.851)	99.930 (9.151)	97.044 (11.998)	<0.001
Mean platelet volume (fL)		9.076 (1.131)	9.062 (1.092)	9.134 (1.273)	0.44
Hemoglobin concentration (g/dL)		138.892 (15.944)	140.085 (15.643)	134.153 (16.289)	<0.001
Platelet distribution width (fL)		15.524 (1.328)	15.461 (0.956)	15.773 (2.255)	0.004
Monocyte percentage (%)		9.376 (1.968)	9.326 (1.874)	9.578 (2.301)	0.121
Platelet count (thousand/μL)		190.254 (60.678)	188.992 (56.275)	195.268 (75.662)	0.211
Monocyte count (thousand/μL)		0.509 (0.173)	0.507 (0.172)	0.517 (0.178)	0.495
Platelet crit rate (%)		0.171 (0.050)	0.169 (0.046)	0.176 (0.062)	0.104
Urine pH (−)		5.346 (0.557)	5.349 (0.560)	5.333 (0.543)	0.739
Serum alanine aminotransferase (U/L)		23.612 (14.569)	23.916 (12.815)	22.404 (20.081)	0.21
Serum aspartate aminotransferase (U/L)		22.079 (16.181)	21.595 (8.587)	24.000 (31.764)	0.072
Total bilirubin (mmol/L)		17.593 (6.754)	17.639 (6.773)	17.407 (6.697)	0.678
Total protein (g/L)		72.119 (5.465)	72.133 (5.239)	72.062 (6.297)	0.874
Albumin (g/L)		43.898 (4.250)	44.128 (4.061)	42.982 (4.835)	0.001
Globulin (g/L)		28.453 (8.338)	28.292 (8.738)	29.093 (6.491)	0.245
Direct bilirubin (mmol/L)		3.354 (1.696)	3.280 (1.555)	3.648 (2.148)	0.009
Serum creatinine (mmol/L)		71.047 (34.713)	70.957 (35.370)	71.404 (32.062)	0.876
Blood urea nitrogen (mmol/L)		5.936 (1.810)	5.865 (1.664)	6.218 (2.286)	0.018
Uric acid (mmol/L)		342.485 (92.540)	345.394 (90.497)	330.929 (99.669)	0.059
Fasting blood glucose (mmol/L)		6.228 (1.956)	6.187 (1.803)	6.389 (2.469)	0.214
Total cholesterol (mmol/L)		4.915 (1.158)	4.928 (1.105)	4.864 (1.351)	0.507
Serum low-density lipoprotein (mmol/L)		2.611 (1.114)	2.648 (1.081)	2.463 (1.229)	0.045
Serum high-density lipoprotein (mmol/L)		1.268 (0.382)	1.261 (0.311)	1.297 (0.586)	0.256
Triglycerides (mmol/L)		1.782 (1.122)	1.699 (0.905)	2.113 (1.698)	<0.001

SD, Standard Deviation; SMI, Skeletal Muscle Index; BMI, Body Mass Index.

### 3.2 Features selecting

**Figure 2:**
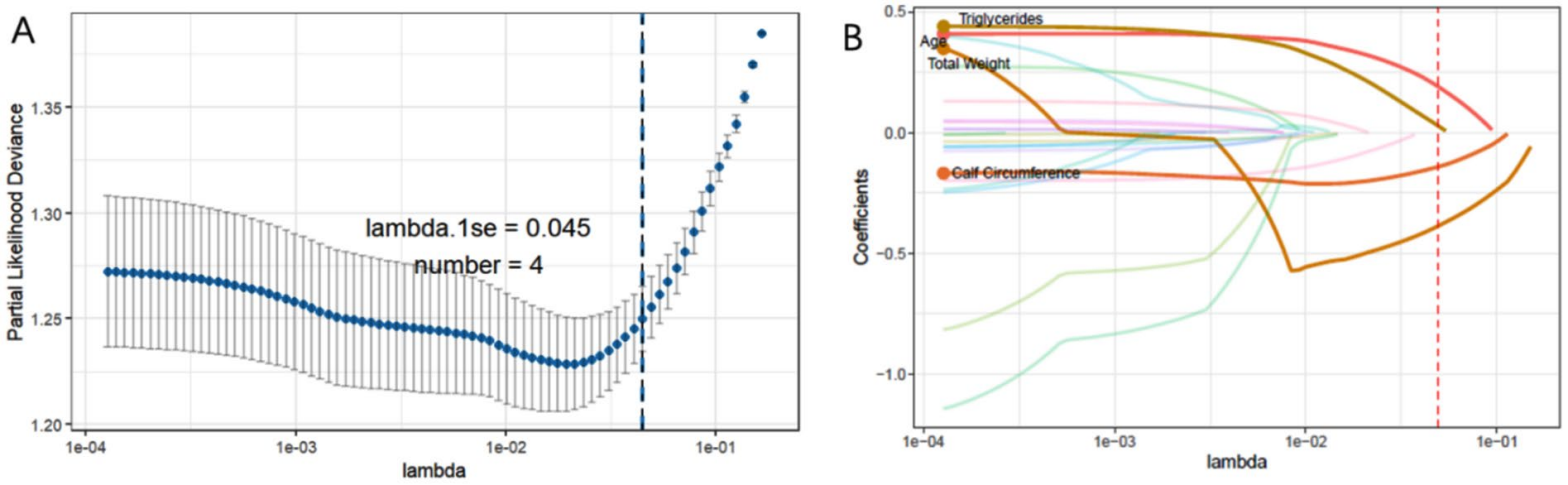
**(A)** The process of selecting the most suitable λ through 10-fold cross-validation in the Lasso model. **(B)** LASSO coefficient profiles.

The Lasso regression with 10-fold cross-validation was trained using distinct features from sarcopenic and non-sarcopenic groups, as shown in Figure 2. The optimal lambda.1se threshold was applied for model selection, yielding four significant predictors: weight, age, calf circumference, and triglycerides.

### 3.3 Model developing and performance evaluating

**Figure 3:**
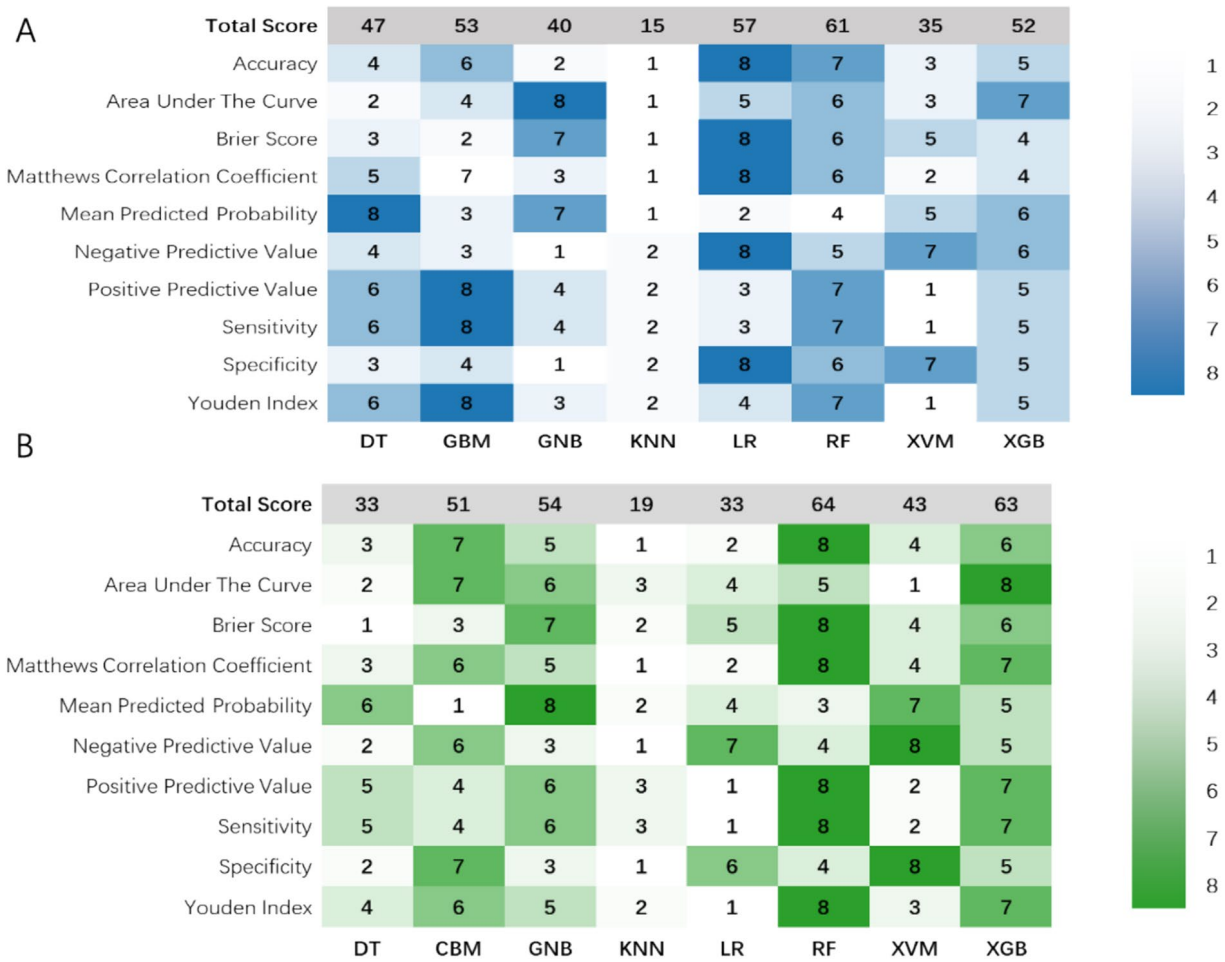
Visualization of the 10 prediction measures using a heat map in the **(A)** internal validation set and **(B)** external validation set after evaluation using a scoring system. DT, Decision Tree; GNB, Gaussian Naïve Bayes; GBM, Light Gradient Boosting Machine; KNN, K-Nearest Neighbors; LR, Logistic Regression; RF, Random Forest; SVM, Support Vector Machine; XGB, Extreme Gradient Boosting.

In the model’s comprehensive scoring system, the Random Forest (RF) showed the best predictive performance in internal validation, with a score of 61 (Figure 3A; Table 2), followed by Logistic Regression (LR) with 57 and Gradient Boosting Machine (GBM) with 54. In the external validation set, the top scores were for RF (64), Extreme Gradient Boosting (XGB) (63), and Gaussian Naïve Bayes (GNB) (37) (Figure 3B; Table 3). Model comparison through ROC curve analysis was shown in Supplementary Figures S1, S2.

**Table 2:** The 10 diagnostic parameters of the eight algorithms in validation set.

Model performance	DT	GBM	GNB	KKNN	LR	RF	SVM	XGB
Accuracy	0.855	0.862	0.841	0.834	0.89	0.869	0.855	0.862
AUC (95% CI)	0.827	0.869	0.879	0.781	0.871	0.872	0.869	0.876
	(0.726, 0.927)	(0.788, 0.95)	(0.8, 0.958)	(0.672, 0.89)	(0.787, 0.954)	(0.793, 0.95)	(0.795, 0.944)	(0.799, 0.953)
Brier score	0.109	0.115	0.099	0.125	0.094	0.101	0.103	0.109
MCC	0.537	0.568	0.492	0.44	0.588	0.566	0.467	0.534
Mean predicted probability	0.221	0.211	0.221	0.187	0.21	0.213	0.215	0.221
NPV	0.884	0.884	0.876	0.884	0.942	0.901	0.917	0.901
PPV	0.708	0.75	0.667	0.583	0.625	0.708	0.542	0.667
Sensitivity	0.708	0.75	0.667	0.583	0.625	0.708	0.542	0.667
Specificity	0.884	0.884	0.876	0.884	0.942	0.901	0.917	0.901
Youden’s index	0.593	0.634	0.543	0.468	0.567	0.609	0.459	0.567

DT, Decision Tree; GNB, Gaussian Naïve Bayes; GBM, Light Gradient Boosting Machine; KKNN, K-Nearest Neighbors; LR, Logistic Regression; RF, Random Forest; SVM, Support Vector Machine; XGB, Extreme Gradient Boosting; AUC, Area Under the Curve; CI, Confidence Interval; MCC, Matthews Correlation Coefficient; PPV, Positive Predictive Value; NPV, Negative Predictive Value.

**Table 3:** The 10 diagnostic parameters of the eight algorithms in test set.

Model performance	DT	GBM	GNB	KKNN	LR	RF	SVM	XGB
Accuracy	0.784	0.805	0.789	0.762	0.773	0.811	0.789	0.805
AUC (95% CI)	0.769	0.855	0.852	0.806	0.812	0.841	0.742	0.865
	(0.695, 0.843)	(0.794, 0.915)	(0.794, 0.909)	(0.737, 0.875)	(0.744, 0.88)	(0.777, 0.904)	(0.654, 0.83)	(0.811, 0.919)
Brier score	0.169	0.167	0.152	0.169	0.157	0.147	0.162	0.155
MCC	0.439	0.492	0.452	0.371	0.390	0.511	0.442	0.493
Mean predicted probability	0.220	0.181	0.223	0.188	0.211	0.209	0.221	0.214
NPV	0.908	0.947	0.916	0.908	0.947	0.931	0.962	0.939
PPV	0.481	0.463	0.481	0.407	0.352	0.519	0.370	0.481
Sensitivity	0.481	0.463	0.481	0.407	0.352	0.519	0.370	0.481
Specificity	0.908	0.947	0.916	0.908	0.947	0.931	0.962	0.939
Youden’s Index	0.390	0.410	0.398	0.316	0.298	0.450	0.332	0.420

DT, Decision Tree; GNB, Gaussian Naïve Bayes; GBM, Light Gradient Boosting Machine; KKNN, K-Nearest Neighbors; LR, Logistic Regression; RF, Random Forest; SVM, Support Vector Machine; XGB, Extreme Gradient Boosting; AUC, Area Under the Curve; CI, Confidence Interval; MCC, Matthews Correlation Coefficient; PPV, Positive Predictive Value; NPV, Negative Predictive Value.

RF and XGB both demonstrated good performance across both datasets, with RF being particularly outstanding in both internal and external validations, making it a top choice as the primary model. LR performs well with linearly separable problems but needs improvement in its generalization to external datasets. Thus, it is considered best to use RF as the baseline model due to its reliable performance and strong generalization capabilities across various datasets. The RF-based predictive model has been set as the optimal model and is named the w-ACT model after the initial letters of the risk factors.

### 3.4 Model explaining

**Figure 4:**
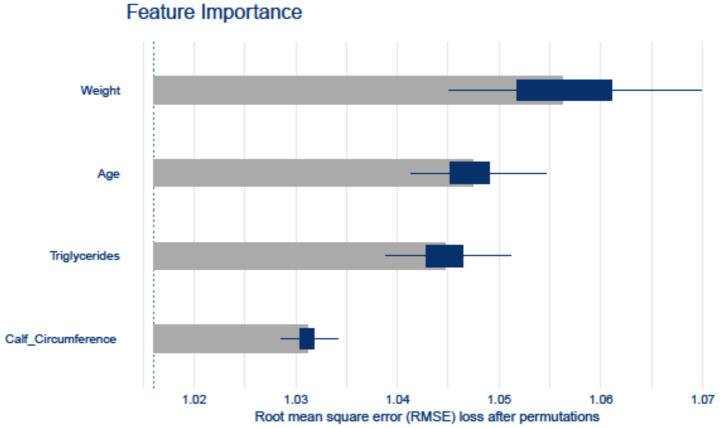
Feature importance computed by the Random Forest model. The horizontal axis represents various features, while the vertical axis represents their respective importance scores.

In the internal validation set, SHAP analysis and permutation importance scoring revealed the hierarchical contribution of w-ACT model features (Figure 4). Weight emerged as the strongest predictor, followed by Age and Triglycerides, with Calf Circumference showing relatively lower but still meaningful contribution. The consistency between SHAP values (directionality) and permutation importance (magnitude) confirmed the model’s clinical plausibility.

## 4 Discussion

Recent research has sought to develop risk prediction models for sarcopenia in community-dwelling older adult populations, facing challenges in data accessibility, interpretability, and predictive accuracy. To address these issues, we utilized local data from the NBPHS program and applied advanced machine learning techniques to create the w-ACT model. Our findings demonstrate that the w-ACT model outperformed traditional LR and other common ML models in both internal and external validations, highlighting its potential to improve the identification of at-risk older adult populations and inform preventive interventions.

We identified a sarcopenia prevalence of approximately 20.11% among community-dwelling older adults in Chongqing, which is notably higher than rates reported in other regions of China utilizing the AWGS 2019 diagnostic criteria—ranging from 14.62% on the Yunnan-Guizhou Plateau to 13.47% in Tianjin (38) and 10.29 to 13.47% in Shanghai (39, 40). The observed discrepancies may be attributed to regional demographic variations. Chongqing, a typical mountainous city with a severe aging population, has an aging rate of 21.87% according to the results of the seventh national census, ranking fifth nationwide and first in the western region (41). In additional, it may be also contributed by the complex interplay of lifestyle and dietary norms characteristic of Chongqing’s residents (42). A pervasive pattern of high oil and salt intake, common among the city’s inhabitants, may be contributing to this disparity. Moreover, nutritional surveys indicate that over 50.5% of the middle-aged and older adult population are overweight or obese. Future research should explore these factors to develop targeted interventions that preserve muscle health and improve the well-being of the aging population.

The w-ACT tool’s design prioritizes immediate clinical utility through three key characteristics. Firstly, seamless integration with existing workflows. The selected indicators (age, weight, CC, TG) are routinely collected in NBPHS service projects, which promote equal access to public health services. The four risk factors—age, weight, CC, and TG—are readily obtainable across various older adult care settings. This allows direct implementation without requiring additional tests or equipment.

Secondly, these risk factors provide significant interpretability. The model’s clinical adoption potential stems from its deliberate avoidance of computationally significant but clinically obscure features - a common criticism of ML healthcare applications. Our four predictors were selected not only for statistical contribution but for their established clinical meaning and measurability in resource-limited settings Consistent with existing guidelines, age, low weight, and CC are established risk factors for sarcopenia, and their non-invasive measurement methods facilitate assessment in diverse settings (1). Notably, CC serves as an index of free fat muscle mass (FFM) and has been recognized by the WHO as a sensitive indicator of muscle mass in older adults (43). Our study also identified triglycerides as a potential risk factor, aligning with previous research (44–46). Researches has established a bidirectional causal relationship between triglycerides and sarcopenia, particularly regarding muscle quality (47, 48). Although the specific mechanisms remain incompletely understood, theories such as the “cycle metabolism theory” suggest that elevated blood lipid levels may contribute to fat accumulation in skeletal muscle, ultimately leading to mitochondrial dysfunction and insulin resistance (49). Thus, the inclusion of these four risk factors enhances both the credibility of the model and facilitates clinical practitioners’ understanding of its predictive foundation.

Thirdly, Despite the advantages of machine learning algorithms, the comparative performance of ML versus traditional LR remains debated (50). While some studies suggest that ML does not consistently outperform LR (51, 52), aligning with the majority of literature (37, 51, 53), our findings indicate superior predictive performance of the ML, especially RF algorithm, which emerged as the top performer among the models assessed. The observed differences may stem from prior research primarily focusing on singular predictive metrics, without considering a comprehensive range of evaluation metrics. Notably, the RF algorithm is praised for its resistance to overfitting, adaptability to categorical variables, accuracy in error rate estimation, and capability of ranking variables by relative importance. These characteristics have led to its successful application in stoke (54), cancer (55), and postoperative functional (56) prediction. Thus, our findings furnish additional evidence for the application of the RF algorithm in risk prediction and serve as a reference for peers in evaluating model performance and selecting algorithms.

Some limitations should be mentioned. First, the cross-sectional NBPHS design precludes causal inference, though we mitigated this via instrumental variable analysis (57). To definitively address these limitations, we will launch the Chongqing Aging and Sarcopenia Evaluation (CHASE) cohort. This prospective study is specifically designed to document predictor trajectories preceding sarcopenia onset and validate dynamic predictions. Second, although internal-external validation was conducted, demonstrating the same level of reliability, the external validation sample size (*n* = 182) may limit the assessment of overfitting risks, particularly given the model’s complexity. While our permutation-based feature importance analysis (Figure 4) shows biologically plausible weightings, the possibility of overfitting to specific subpopulations cannot be entirely excluded. Additionally, dealing with imbalanced data is a challenging issue in both deep learning models and traditional models for practical classification problems (58). While oversampling improved model training, its theoretical impact on population prevalence estimates cannot be fully excluded - though our external validation using original unbalanced data suggests robust generalizability. Third, while our model demonstrated consistent performance across internal and external sets, its generalizability to populations with different ethnic compositions or healthcare systems requires further validation, particularly given regional variations in sarcopenia prevalence and risk factors. Normalization assumptions may not perfectly fit all variables, but this approach represents the most widely accepted compromise for mixed-type clinical data (59, 60).

## 5 Conclusion

This study developed a ML-based predictive model to identify early-stage high-risk individuals for sarcopenia among older adult residents in the community. Weight, triglycerides, age, and calf circumference were identified as significant factors associated with sarcopenia, and RF exhibited superior predictive performance in eight approaches. The interpretability of this model (termed w-ACT model) was achieved using SHAP values, providing valuable insights into the contributions of these variables to sarcopenia risk.

## Data Availability

The raw data supporting the conclusions of this article will be made available by the authors, upon reasonable request.
