# Supplementary material for: A w-ACT model for sarcopenia among community-dwelling older adults based on National Basic Public Health Services: development and validation study
Source: Front Public Health. 2025 Aug 26;13:1522903. doi: 10.3389/fpubh.2025.1522903 (PMC12419224; doi:10.3389/fpubh.2025.1522903)
Supplement: Supplementary file 1 [file Data_Sheet_1.docx]

**Table S1 Demographic and clinical characteristics of training set (n=580)**

| **Variables** | **Categories** | **Overall (n=580)** | **Non-sarcopenia (n=475)** | **Sarcopenia (n=105)** | **P-value** |
| --- | --- | --- | --- | --- | --- |
| Gender (%) | Female | 326 (56.2) | 260 (54.7) | 66 (62.9) | 0.159 |
|  | Male | 254 (43.8) | 215 (45.3) | 39 (37.1) |  |
| Education Level (%) | Illiterate | 58 (10.0) | 40 (8.4) | 18 (17.1) | 0.018 |
|  | Elementary education | 84 (14.5) | 66 (13.9) | 18 (17.1) |  |
|  | Junior school education | 234 (40.3) | 202 (42.5) | 32 (30.5) |  |
|  | High school education | 119 (20.5) | 94 (19.8) | 25 (23.8) |  |
|  | Tertiary education | 85 (14.7) | 73 (15.4) | 12 (11.4) |  |
| Marital Status (%) | Married | 459 (79.1) | 380 (80.0) | 79 (75.2) | 0.447 |
|  | Divorced | 15 (2.6) | 13 (2.7) | 2 (1.9) |  |
|  | Widowed | 68 (11.7) | 51 (10.7) | 17 (16.2) |  |
|  | Other/ Prefer not to answer | 38 (6.6) | 31 (6.5) | 7 (6.7) |  |
| Employment Status (%) | Retired | 558 (96.2) | 456 (96.0) | 102 (97.1) | 0.785 |
|  | Engaged in farming/work | 22 (3.8) | 19 (4.0) | 3 (2.9) |  |
| Living Situation (%) | Living alone | 84 (14.5) | 69 (14.5) | 15 (14.3) | 0.225 |
|  | Living with spouse | 308 (53.1) | 260 (54.7) | 48 (45.7) |  |
|  | Living with children | 70 (12.1) | 52 (10.9) | 18 (17.1) |  |
|  | Living with spouse and children | 100 (17.2) | 78 (16.4) | 22 (21.0) |  |
|  | Others | 18 (3.1) | 16 (3.4) | 2 (1.9) |  |
| Medical Insurance Status (%) | Resident medical insurance | 75 (12.9) | 59 (12.4) | 16 (15.2) | 0.306 |
|  | Employee medical insurance | 475 (81.9) | 394 (82.9) | 81 (77.1) |  |
|  | Others | 30 (5.2) | 22 (4.6) | 8 (7.6) |  |
| Overall Assessment of Your Health Status (%) | Very poor | 46 (7.9) | 36 (7.6) | 10 (9.5) | 0.006 |
|  | Relatively poor | 209 (36.0) | 157 (33.1) | 52 (49.5) |  |
|  | Relatively good | 285 (49.1) | 246 (51.8) | 39 (37.1) |  |
|  | Very good | 40 (6.9) | 36 (7.6) | 4 (3.8) |  |
| Traditional Chinese Medicine Constitution (%) | Balanced constitution | 104 (17.9) | 82 (17.3) | 22 (21.0) | <0.001 |
|  | Dampness-heat constitution | 50 (8.6) | 32 (6.7) | 18 (17.1) |  |
|  | Phlegm-dampness constitution | 105 (18.1) | 86 (18.1) | 19 (18.1) |  |
|  | Qi-deficiency constitution | 144 (24.8) | 140 (29.5) | 4 (3.8) |  |
|  | Yang-deficiency constitution | 172 (29.7) | 131 (27.6) | 41 (39.0) |  |
|  | Yin-deficiency constitution | 5 (0.9) | 4 (0.8) | 1 (1.0) |  |
| Average Sleep Time in the Past Month (%) | <6 hours | 233 (40.2) | 189 (39.8) | 44 (41.9) | 0.31 |
|  | 6~8 hours | 249 (42.9) | 210 (44.2) | 39 (37.1) |  |
|  | >8 hours | 98 (16.9) | 76 (16.0) | 22 (21.0) |  |
| Average Daily Step Count in the Last Three Days (%) | <2000 steps | 74 (12.8) | 54 (11.4) | 20 (19.0) | 0.017 |
|  | 2000~4000 steps | 99 (17.1) | 73 (15.4) | 26 (24.8) |  |
|  | 4000~6000 steps | 137 (23.6) | 115 (24.2) | 22 (21.0) |  |
|  | 6000~8000 steps | 98 (16.9) | 81 (17.1) | 17 (16.2) |  |
|  | 8000~10000 steps | 39 (6.7) | 35 (7.4) | 4 (3.8) |  |
|  | >10000 steps | 133 (22.9) | 117 (24.6) | 16 (15.2) |  |
| Hypertension (%) | No | 163 (28.1) | 139 (29.3) | 24 (22.9) | 0.23 |
|  | Yes | 417 (71.9) | 336 (70.7) | 81 (77.1) |  |
| Diabetes (%) | No | 363 (62.6) | 298 (62.7) | 65 (61.9) | 0.962 |
|  | Yes | 217 (37.4) | 177 (37.3) | 40 (38.1) |  |
| Smoking in the Past Three Months (%) | No | 516 (89.0) | 419 (88.2) | 97 (92.4) | 0.288 |
|  | Yes | 64 (11.0) | 56 (11.8) | 8 (7.6) |  |
| Drinking Alcohol in the Past Three Months (%) | No | 521 (89.8) | 422 (88.8) | 99 (94.3) | 0.136 |
|  | Yes | 59 (10.2) | 53 (11.2) | 6 (5.7) |  |
| Pain Symptoms in the Past Three Months (%) | No | 550 (94.8) | 453 (95.4) | 97 (92.4) | 0.314 |
|  | Yes | 30 (5.2) | 22 (4.6) | 8 (7.6) |  |
| Falls in the Past Year (%) | No | 504 (86.9) | 414 (87.2) | 90 (85.7) | 0.813 |
|  | Yes | 76 (13.1) | 61 (12.8) | 15 (14.3) |  |
| Grip Strength (Kg, SD) | | 24.547 (7.806) | 25.987 (7.609) | 18.030 (4.794) | <0.001 |
| SMI (Kg/m^2^ ,SD) | | 6.494 (1.622) | 6.723 (1.678) | 5.455 (0.694) | <0.001 |
| Age |  | 71.684 (5.633) | 70.985 (5.080) | 74.848 (6.831) | <0.001 |
| Height (cm) |  | 156.091 (8.563) | 157.232 (8.336) | 150.933 (7.658) | <0.001 |
| Total Weight (Kg) | | 59.907 (9.637) | 61.841 (9.073) | 51.157 (6.891) | <0.001 |
| Waist Circumference (cm) | | 83.902 (9.769) | 85.032 (9.794) | 78.790 (7.871) | <0.001 |
| Calf Circumference (cm) | | 33.871 (2.823) | 34.385 (2.659) | 31.543 (2.337) | <0.001 |
| BMI (kg/m^2^) |  | 24.543 (3.307) | 25.001 (3.261) | 22.469 (2.663) | <0.001 |
| Waist-Hip Ratio (-) | | 0.883 (0.057) | 0.890 (0.057) | 0.852 (0.050) | <0.001 |
| White Blood Cell Count (cells/μL) | | 5.394 (1.291) | 5.409 (1.302) | 5.322 (1.241) | 0.533 |
| Red Blood Cells (million/μL) | | 4.919 (0.558) | 4.947 (0.556) | 4.794 (0.556) | 0.011 |
| Red Cell Distribution Width Coefficient of Variation (-) | | 14.043 (1.036) | 14.004 (1.037) | 14.216 (1.016) | 0.058 |
| Hematocrit (%) | | 49.905 (5.394) | 50.216 (5.477) | 48.500 (4.777) | 0.003 |
| Lymphocyte Percentage (μm) | | 31.202 (7.026) | 31.309 (7.044) | 30.715 (6.959) | 0.434 |
| Red Cell Distribution Width Standard Deviation (μm) | | 53.475 (4.733) | 53.376 (4.554) | 53.922 (5.472) | 0.285 |
| Lymphocyte Count (thousand/μL) | | 1.658 (0.468) | 1.669 (0.468) | 1.609 (0.469) | 0.23 |
| Mean Corpuscular Hemoglobin Content (pg/cell) | | 28.913 (2.769) | 28.989 (2.754) | 28.570 (2.824) | 0.161 |
| Neutrophil Percentage (%) | | 59.273 (7.621) | 59.190 (7.700) | 59.648 (7.272) | 0.578 |
| Mean Corpuscular Hemoglobin Concentration (g/L) | | 283.783 (18.983) | 284.368 (19.777) | 281.133 (14.663) | 0.114 |
| Neutrophil Count (thousand/μL) | | 3.267 (1.248) | 3.278 (1.306) | 3.215 (0.940) | 0.641 |
| Mean Corpuscular Volume (fL) | | 101.957 (8.420) | 102.127 (8.059) | 101.187 (9.896) | 0.301 |
| Mean Platelet Volume (fL) | | 8.973 (0.949) | 8.992 (0.955) | 8.890 (0.923) | 0.324 |
| Hemoglobin Concentration (g/dL) | | 141.684 (14.686) | 142.838 (14.624) | 136.467 (13.873) | <0.001 |
| Platelet Distribution Width (fL) | | 15.458 (0.437) | 15.462 (0.467) | 15.442 (0.268) | 0.669 |
| Monocyte Percentage (%) | | 9.447 (1.829) | 9.405 (1.778) | 9.637 (2.041) | 0.239 |
| Platelet Count (thousand/μL) | | 188.564 (54.784) | 187.554 (54.562) | 193.133 (55.816) | 0.345 |
| Monocyte Count (thousand/μL) | | 0.492 (0.156) | 0.492 (0.155) | 0.494 (0.162) | 0.882 |
| Platelet Crit Rate (%) | | 0.167 (0.043) | 0.167 (0.043) | 0.170 (0.045) | 0.461 |
| Urine pH (-) |  | 5.368 (0.575) | 5.361 (0.568) | 5.400 (0.610) | 0.531 |
| Serum Alanine Aminotransferase (U/L) | | 23.600 (11.797) | 23.888 (11.575) | 22.295 (12.726) | 0.211 |
| Serum Aspartate Aminotransferase (U/L) | | 21.141 (7.824) | 21.007 (7.083) | 21.743 (10.570) | 0.384 |
| Total Bilirubin (mmol/L) | | 18.220 (6.724) | 18.219 (6.792) | 18.225 (6.439) | 0.994 |
| Total Protein (g/L) | | 72.534 (5.179) | 72.746 (5.069) | 71.576 (5.576) | 0.036 |
| Albumin (g/L) | | 44.907 (3.757) | 45.003 (3.657) | 44.469 (4.168) | 0.187 |
| Globulin (g/L) |  | 27.984 (9.492) | 28.178 (10.210) | 27.108 (5.062) | 0.296 |
| Direct Bilirubin (mmol/L) | | 3.207 (1.464) | 3.171 (1.419) | 3.369 (1.654) | 0.211 |
| Serum Creatinine (mmol/L) | | 69.770 (19.324) | 70.184 (18.120) | 67.895 (24.047) | 0.272 |
| Blood Urea Nitrogen (mmol/L) | | 5.825 (1.613) | 5.844 (1.562) | 5.736 (1.832) | 0.533 |
| Uric Acid (mmol/L) | | 346.393 (90.653) | 350.057 (90.218) | 329.819 (91.195) | 0.038 |
| Fasting Blood Glucose (mmol/L) | | 6.194 (1.916) | 6.189 (1.818) | 6.220 (2.318) | 0.881 |
| Total Cholesterol (mmol/L) | | 5.014 (1.162) | 5.027 (1.082) | 4.955 (1.474) | 0.57 |
| Serum Low-Density Lipoprotein (mmol/L) | | 2.964 (0.948) | 2.981 (0.915) | 2.887 (1.084) | 0.362 |
| Serum High-Density Lipoprotein (mmol/L) | | 1.332 (0.420) | 1.310 (0.316) | 1.431 (0.719) | 0.007 |
| Triglycerides (mmol/L) | | 1.591 (1.123) | 1.519 (0.808) | 1.917 (1.978) | 0.001 |

**Abbreviations:** SD: Standard Deviation. SMI: Skeletal Muscle Index. BMI: Body Mass Index

**Table S2 Demographic and clinical characteristics of validation set (n=145)**

| **Variables** | **Categories** | **Overall (n=145)** | **Non-sarcopenia (n=121)** | **Sarcopenia (n=24)** | **P-value** |
| --- | --- | --- | --- | --- | --- |
| Gender (%) | Female | 88 (60.7) | 77 (63.6) | 11 (45.8) | 0.161 |
|  | Male | 57 (39.3) | 44 (36.4) | 13 (54.2) |  |
| Education Level (%) | Illiterate | 16 (11.0) | 13 (10.7) | 3 (12.5) | 0.462 |
|  | Elementary education | 16 (11.0) | 11 (9.1) | 5 (20.8) |  |
|  | Junior school education | 60 (41.4) | 53 (43.8) | 7 (29.2) |  |
|  | High school education | 29 (20.0) | 24 (19.8) | 5 (20.8) |  |
|  | Tertiary education | 24 (16.6) | 20 (16.5) | 4 (16.7) |  |
| Marital Status (%) | Married | 104 (71.7) | 88 (72.7) | 16 (66.7) | 0.815 |
|  | Divorced | 1 (0.7) | 1 (0.8) | 0 (0.0) |  |
|  | Widowed | 27 (18.6) | 21 (17.4) | 6 (25.0) |  |
|  | Other/ Prefer not to answer | 13 (9.0) | 11 (9.1) | 2 (8.3) |  |
| Employment Status (%) | Retired | 136 (93.8) | 112 (92.6) | 24 (100.0) | 0.359 |
|  | Engaged in farming/work | 9 (6.2) | 9 (7.4) | 0 (0.0) |  |
| Living Situation (%) | Living alone | 35 (24.1) | 28 (23.1) | 7 (29.2) | 0.801 |
|  | Living with spouse | 68 (46.9) | 56 (46.3) | 12 (50.0) |  |
|  | Living with children | 18 (12.4) | 15 (12.4) | 3 (12.5) |  |
|  | Living with spouse and children | 22 (15.2) | 20 (16.5) | 2 (8.3) |  |
|  | Others | 2 (1.4) | 2 (1.7) | 0 (0.0) |  |
| Medical Insurance Status (%) | Resident medical insurance | 17 (11.7) | 12 (9.9) | 5 (20.8) | 0.208 |
|  | Employee medical insurance | 123 (84.8) | 104 (86.0) | 19 (79.2) |  |
|  | Others | 5 (3.4) | 5 (4.1) | 0 (0.0) |  |
| Overall Assessment of Your Health Status (%) | Very poor | 10 (6.9) | 7 (5.8) | 3 (12.5) | 0.459 |
|  | Relatively poor | 52 (35.9) | 46 (38.0) | 6 (25.0) |  |
|  | Relatively good | 75 (51.7) | 61 (50.4) | 14 (58.3) |  |
|  | Very good | 8 (5.5) | 7 (5.8) | 1 (4.2) |  |
| Traditional Chinese Medicine Constitution (%) | Balanced constitution | 24 (16.6) | 19 (15.7) | 5 (20.8) | 0.118 |
|  | Dampness-heat constitution | 14 (9.7) | 10 (8.3) | 4 (16.7) |  |
|  | Phlegm-dampness constitution | 25 (17.2) | 20 (16.5) | 5 (20.8) |  |
|  | Qi-deficiency constitution | 40 (27.6) | 39 (32.2) | 1 (4.2) |  |
|  | Yang-deficiency constitution | 41 (28.3) | 32 (26.4) | 9 (37.5) |  |
|  | Yin-deficiency constitution | 1 (0.7) | 1 (0.8) | 0 (0.0) |  |
| Average Sleep Time in the Past Month (%) | <6 hours | 55 (37.9) | 49 (40.5) | 6 (25.0) | 0.041 |
|  | 6~8 hours | 62 (42.8) | 53 (43.8) | 9 (37.5) |  |
|  | >8 hours | 28 (19.3) | 19 (15.7) | 9 (37.5) |  |
| Average Daily Step Count in the Last Three Days (%) | <2000 steps | 27 (18.6) | 21 (17.4) | 6 (25.0) | 0.764 |
|  | 2000~4000 steps | 27 (18.6) | 22 (18.2) | 5 (20.8) |  |
|  | 4000~6000 steps | 32 (22.1) | 27 (22.3) | 5 (20.8) |  |
|  | 6000~8000 steps | 23 (15.9) | 19 (15.7) | 4 (16.7) |  |
|  | 8000~10000 steps | 9 (6.2) | 9 (7.4) | 0 (0.0) |  |
|  | >10000 steps | 27 (18.6) | 23 (19.0) | 4 (16.7) |  |
| Hypertension (%) | No | 43 (29.7) | 37 (30.6) | 6 (25.0) | 0.763 |
|  | Yes | 102 (70.3) | 84 (69.4) | 18 (75.0) |  |
| Diabetes (%) | No | 89 (61.4) | 75 (62.0) | 14 (58.3) | 0.916 |
|  | Yes | 56 (38.6) | 46 (38.0) | 10 (41.7) |  |
| Smoking in the Past Three Months (%) | No | 131 (90.3) | 110 (90.9) | 21 (87.5) | 0.89 |
|  | Yes | 14 (9.7) | 11 (9.1) | 3 (12.5) |  |
| Drinking Alcohol in the Past Three Months (%) | No | 129 (89.0) | 107 (88.4) | 22 (91.7) | 0.916 |
|  | Yes | 16 (11.0) | 14 (11.6) | 2 (8.3) |  |
| Pain Symptoms in the Past Three Months (%) | No | 136 (93.8) | 112 (92.6) | 24 (100.0) | 0.359 |
|  | Yes | 9 (6.2) | 9 (7.4) | 0 (0.0) |  |
| Falls in the Past Year (%) | No | 130 (89.7) | 112 (92.6) | 18 (75.0) | 0.027 |
|  | Yes | 15 (10.3) | 9 (7.4) | 6 (25.0) |  |
| Grip Strength (Kg, SD) | | 24.271 (8.048) | 25.327 (7.999) | 18.946 (6.016) | <0.001 |
| SMI (Kg/m^2^ ,SD) | | 6.385 (0.963) | 6.678 (0.858) | 5.674 (0.830) | <0.001 |
| Age |  | 72.069 (6.601) | 70.983 (5.607) | 77.542 (8.449) | <0.001 |
| Height (cm) |  | 156.179 (8.756) | 156.512 (8.433) | 154.500 (10.269) | 0.305 |
| Total Weight (Kg) | | 60.097 (9.784) | 61.827 (9.286) | 51.375 (7.369) | <0.001 |
| Waist Circumference (cm) | | 82.959 (9.351) | 84.074 (9.391) | 77.333 (6.914) | 0.001 |
| Calf Circumference (cm) | | 33.938 (3.083) | 34.550 (2.837) | 30.854 (2.375) | <0.001 |
| BMI (kg/m^2^) |  | 24.670 (3.258) | 25.302 (3.105) | 21.488 (1.865) | <0.001 |
| Waist-Hip Ratio (-) | | 0.884 (0.058) | 0.890 (0.059) | 0.852 (0.044) | 0.003 |
| White Blood Cell Count (cells/μL) | | 5.391 (1.358) | 5.433 (1.315) | 5.177 (1.574) | 0.401 |
| Red Blood Cells (million/μL) | | 4.890 (0.521) | 4.901 (0.494) | 4.832 (0.649) | 0.555 |
| Red Cell Distribution Width Coefficient of Variation (-) | | 14.000 (0.862) | 14.017 (0.890) | 13.912 (0.719) | 0.588 |
| Hematocrit (%) | | 49.851 (4.940) | 49.872 (4.657) | 49.746 (6.299) | 0.91 |
| Lymphocyte Percentage (μm) | | 30.879 (7.290) | 30.605 (7.308) | 32.258 (7.188) | 0.312 |
| Red Cell Distribution Width Standard Deviation (μm) | | 53.497 (4.390) | 53.437 (4.460) | 53.800 (4.093) | 0.713 |
| Lymphocyte Count (thousand/μL) | | 1.646 (0.519) | 1.651 (0.518) | 1.621 (0.530) | 0.794 |
| Mean Corpuscular Hemoglobin Content (pg/cell) | | 28.862 (2.094) | 28.832 (2.048) | 29.012 (2.355) | 0.702 |
| Neutrophil Percentage (%) | | 59.688 (7.724) | 60.017 (7.838) | 58.029 (7.036) | 0.251 |
| Mean Corpuscular Hemoglobin Concentration (g/L) | | 282.986 (16.424) | 283.165 (17.456) | 282.083 (9.904) | 0.769 |
| Neutrophil Count (thousand/μL) | | 3.257 (1.022) | 3.293 (1.003) | 3.071 (1.119) | 0.332 |
| Mean Corpuscular Volume (fL) | | 101.604 (7.883) | 101.974 (6.024) | 99.742 (13.977) | 0.206 |
| Mean Platelet Volume (fL) | | 8.953 (0.981) | 9.026 (0.921) | 8.583 (1.197) | 0.043 |
| Hemoglobin Concentration (g/dL) | | 140.917 (13.486) | 140.975 (12.435) | 140.625 (18.216) | 0.908 |
| Platelet Distribution Width (fL) | | 15.550 (0.351) | 15.550 (0.351) | 15.550 (0.360) | 0.996 |
| Monocyte Percentage (%) | | 9.479 (1.891) | 9.355 (1.746) | 10.104 (2.449) | 0.076 |
| Platelet Count (thousand/μL) | | 185.497 (56.207) | 184.884 (56.197) | 188.583 (57.364) | 0.77 |
| Monocyte Count (thousand/μL) | | 0.495 (0.160) | 0.494 (0.156) | 0.500 (0.184) | 0.872 |
| Platelet Crit Rate (%) | | 0.164 (0.043) | 0.165 (0.043) | 0.159 (0.042) | 0.549 |
| Urine pH (-) |  | 5.414 (0.611) | 5.463 (0.640) | 5.167 (0.351) | 0.029 |
| Serum Alanine Aminotransferase (U/L) | | 22.614 (11.058) | 23.281 (11.534) | 19.250 (7.571) | 0.103 |
| Serum Aspartate Aminotransferase (U/L) | | 20.848 (7.196) | 21.008 (7.065) | 20.042 (7.937) | 0.55 |
| Total Bilirubin (mmol/L) | | 17.688 (5.860) | 17.707 (6.020) | 17.587 (5.089) | 0.927 |
| Total Protein (g/L) | | 72.481 (5.044) | 72.079 (4.801) | 74.504 (5.823) | 0.031 |
| Albumin (g/L) | | 45.207 (3.321) | 45.163 (3.185) | 45.429 (4.005) | 0.721 |
| Globulin (g/L) |  | 27.272 (4.196) | 26.883 (4.005) | 29.231 (4.659) | 0.012 |
| Direct Bilirubin (mmol/L) | | 3.197 (1.433) | 3.234 (1.507) | 3.008 (0.976) | 0.483 |
| Serum Creatinine (mmol/L) | | 70.862 (16.684) | 69.537 (16.156) | 77.542 (18.029) | 0.031 |
| Blood Urea Nitrogen (mmol/L) | | 5.923 (1.512) | 5.826 (1.455) | 6.416 (1.717) | 0.081 |
| Uric Acid (mmol/L) | | 348.903 (84.603) | 345.802 (82.977) | 364.542 (92.654) | 0.323 |
| Fasting Blood Glucose (mmol/L) | | 6.005 (1.403) | 5.921 (1.223) | 6.430 (2.075) | 0.104 |
| Total Cholesterol (mmol/L) | | 4.988 (1.051) | 4.917 (1.069) | 5.350 (0.888) | 0.065 |
| Serum Low-Density Lipoprotein (mmol/L) | | 2.945 (0.923) | 2.906 (0.927) | 3.141 (0.894) | 0.257 |
| Serum High-Density Lipoprotein (mmol/L) | | 1.316 (0.288) | 1.312 (0.292) | 1.337 (0.269) | 0.698 |
| Triglycerides (mmol/L) | | 1.559 (0.934) | 1.441 (0.663) | 2.158 (1.650) | <0.001 |

**Abbreviations:** SD: Standard Deviation. SMI: Skeletal Muscle Index. BMI: Body Mass Index

**Table S3 Demographic and clinical characteristics of test set (n=185)**

| **Variables** | **Categories** | **Overall (n=185)** | **Non-sarcopenia (n=131)** | **Sarcopenia (n=54)** | **P-value** |
| --- | --- | --- | --- | --- | --- |
| n |  | 185 | 131 | 54 |  |
| Gender (%) | Female | 94 (50.8) | 68 (51.9) | 26 (48.1) | 0.762 |
|  | Male | 91 (49.2) | 63 (48.1) | 28 (51.9) |  |
| Education Level (%) | Illiterate | 29 (15.7) | 23 (17.6) | 6 (11.1) | 0.355 |
|  | Elementary education | 40 (21.6) | 25 (19.1) | 15 (27.8) |  |
|  | Junior school education | 81 (43.8) | 55 (42.0) | 26 (48.1) |  |
|  | High school education | 27 (14.6) | 21 (16.0) | 6 (11.1) |  |
|  | Tertiary education | 8 (4.3) | 7 (5.3) | 1 (1.9) |  |
| Marital Status (%) | Married | 140 (75.7) | 101 (77.1) | 39 (72.2) | 0.269 |
|  | Divorced | 1 (0.5) | 1 (0.8) | 0 (0.0) |  |
|  | Widowed | 35 (18.9) | 21 (16.0) | 14 (25.9) |  |
|  | Other/ Prefer not to answer | 9 (4.9) | 8 (6.1) | 1 (1.9) |  |
| Employment Status (%) | Retired | 145 (78.4) | 103 (78.6) | 42 (77.8) | 1 |
|  | Engaged in farming/work | 40 (21.6) | 28 (21.4) | 12 (22.2) |  |
| Living Situation (%) | Living alone | 20 (10.8) | 16 (12.2) | 4 (7.4) | 0.103 |
|  | Living with spouse | 81 (43.8) | 57 (43.5) | 24 (44.4) |  |
|  | Living with children | 38 (20.5) | 21 (16.0) | 17 (31.5) |  |
|  | Living with spouse and children | 37 (20.0) | 29 (22.1) | 8 (14.8) |  |
|  | Others | 9 (4.9) | 8 (6.1) | 1 (1.9) |  |
| Medical Insurance Status (%) | Resident medical insurance | 43 (23.2) | 33 (25.2) | 10 (18.5) | 0.029 |
|  | Employee medical insurance | 106 (57.3) | 79 (60.3) | 27 (50.0) |  |
|  | Others | 36 (19.5) | 19 (14.5) | 17 (31.5) |  |
| Overall Assessment of Your Health Status (%) | Very poor | 25 (13.5) | 16 (12.2) | 9 (16.7) | 0.223 |
|  | Relatively poor | 93 (50.3) | 63 (48.1) | 30 (55.6) |  |
|  | Relatively good | 60 (32.4) | 45 (34.4) | 15 (27.8) |  |
|  | Very good | 7 (3.8) | 7 (5.3) | 0 (0.0) |  |
| Traditional Chinese Medicine Constitution (%) | Balanced constitution | 27 (14.6) | 16 (12.2) | 11 (20.4) | 0.136 |
|  | Dampness-heat constitution | 11 (5.9) | 9 (6.9) | 2 (3.7) |  |
|  | Phlegm-dampness constitution | 21 (11.4) | 14 (10.7) | 7 (13.0) |  |
|  | Qi-deficiency constitution | 59 (31.9) | 48 (36.6) | 11 (20.4) |  |
|  | Yang-deficiency constitution | 64 (34.6) | 41 (31.3) | 23 (42.6) |  |
|  | Yin-deficiency constitution | 3 (1.6) | 3 (2.3) | 0 (0.0) |  |
| Average Sleep Time in the Past Month (%) | <6 hours | 61 (33.0) | 42 (32.1) | 19 (35.2) | 0.249 |
|  | 6~8 hours | 97 (52.4) | 73 (55.7) | 24 (44.4) |  |
|  | >8 hours | 27 (14.6) | 16 (12.2) | 11 (20.4) |  |
| Average Daily Step Count in the Last Three Days (%) | <2000 steps | 55 (29.7) | 36 (27.5) | 19 (35.2) | 0.135 |
|  | 2000~4000 steps | 38 (20.5) | 24 (18.3) | 14 (25.9) |  |
|  | 4000~6000 steps | 24 (13.0) | 20 (15.3) | 4 (7.4) |  |
|  | 6000~8000 steps | 38 (20.5) | 25 (19.1) | 13 (24.1) |  |
|  | 8000~10000 steps | 15 (8.1) | 12 (9.2) | 3 (5.6) |  |
|  | >10000 steps | 15 (8.1) | 14 (10.7) | 1 (1.9) |  |
| Hypertension (%) | No | 132 (71.4) | 91 (69.5) | 41 (75.9) | 0.481 |
|  | Yes | 53 (28.6) | 40 (30.5) | 13 (24.1) |  |
| Diabetes (%) | No | 159 (85.9) | 111 (84.7) | 48 (88.9) | 0.612 |
|  | Yes | 26 (14.1) | 20 (15.3) | 6 (11.1) |  |
| Smoking in the Past Three Months (%) | No | 147 (79.5) | 104 (79.4) | 43 (79.6) | 1 |
|  | Yes | 38 (20.5) | 27 (20.6) | 11 (20.4) |  |
| Drinking Alcohol in the Past Three Months (%) | No | 155 (83.8) | 107 (81.7) | 48 (88.9) | 0.322 |
|  | Yes | 30 (16.2) | 24 (18.3) | 6 (11.1) |  |
| Pain Symptoms in the Past Three Months (%) | No | 170 (91.9) | 122 (93.1) | 48 (88.9) | 0.506 |
|  | Yes | 15 (8.1) | 9 (6.9) | 6 (11.1) |  |
| Falls in the Past Year (%) | No | 157 (84.9) | 114 (87.0) | 43 (79.6) | 0.294 |
|  | Yes | 28 (15.1) | 17 (13.0) | 11 (20.4) |  |
| Grip Strength (Kg, SD) | | 21.850 (8.051) | 23.662 (8.121) | 17.456 (5.958) | <0.001 |
| SMI (Kg/m^2^ ,SD) | | 6.385 (0.963) | 6.678 (0.858) | 5.674 (0.830) | <0.001 |
| Age |  | 71.686 (5.065) | 71.321 (4.779) | 72.574 (5.649) | 0.126 |
| Height (cm) |  | 157.249 (8.147) | 158.057 (8.105) | 155.287 (7.985) | 0.035 |
| Total Weight (Kg) | | 59.059 (9.316) | 61.843 (8.620) | 52.306 (7.311) | <0.001 |
| Waist Circumference (cm) | | 86.800 (9.823) | 88.473 (10.244) | 82.741 (7.339) | <0.001 |
| Calf Circumference (cm) | | 34.224 (5.005) | 35.140 (4.788) | 32.000 (4.860) | <0.001 |
| BMI (kg/m^2^) |  | 23.892 (3.483) | 24.792 (3.326) | 21.707 (2.846) | <0.001 |
| Waist-Hip Ratio (-) | | 0.894 (0.060) | 0.899 (0.059) | 0.882 (0.061) | 0.077 |
| White Blood Cell Count (cells/μL) | | 6.077 (1.787) | 6.091 (1.854) | 6.044 (1.626) | 0.873 |
| Red Blood Cells (million/μL) | | 4.458 (0.646) | 4.454 (0.661) | 4.466 (0.614) | 0.914 |
| Red Cell Distribution Width Coefficient of Variation (-) | | 13.720 (1.236) | 13.563 (1.107) | 14.100 (1.445) | 0.007 |
| Hematocrit (%) | | 42.480 (30.569) | 40.356 (6.686) | 47.633 (55.645) | 0.141 |
| Lymphocyte Percentage (μm) | | 27.212 (8.764) | 27.669 (9.154) | 26.105 (7.704) | 0.271 |
| Red Cell Distribution Width Standard Deviation (μm) | | 51.295 (9.482) | 52.318 (9.514) | 48.813 (9.013) | 0.022 |
| Lymphocyte Count (thousand/μL) | | 1.589 (0.546) | 1.600 (0.566) | 1.564 (0.499) | 0.689 |
| Mean Corpuscular Hemoglobin Content (pg/cell) | | 29.011 (2.825) | 29.205 (2.569) | 28.539 (3.346) | 0.145 |
| Neutrophil Percentage (%) | | 62.776 (9.946) | 62.378 (9.961) | 63.741 (9.937) | 0.398 |
| Mean Corpuscular Hemoglobin Concentration (g/L) | | 324.178 (19.186) | 323.901 (19.497) | 324.852 (18.572) | 0.76 |
| Neutrophil Count (thousand/μL) | | 3.900 (1.592) | 3.909 (1.703) | 3.879 (1.298) | 0.906 |
| Mean Corpuscular Volume (fL) | | 89.410 (9.109) | 90.078 (8.834) | 87.791 (9.636) | 0.121 |
| Mean Platelet Volume (fL) | | 9.495 (1.582) | 9.348 (1.566) | 9.852 (1.579) | 0.049 |
| Hemoglobin Concentration (g/dL) | | 128.551 (17.308) | 129.282 (17.263) | 126.778 (17.449) | 0.372 |
| Platelet Distribution Width (fL) | | 15.710 (2.822) | 15.377 (2.044) | 16.517 (4.057) | 0.012 |
| Monocyte Percentage (%) | | 9.076 (2.388) | 9.012 (2.267) | 9.229 (2.674) | 0.575 |
| Platelet Count (thousand/μL) | | 199.281 (78.508) | 198.000 (61.734) | 202.389 (109.709) | 0.731 |
| Monocyte Count (thousand/μL) | | 0.573 (0.215) | 0.575 (0.223) | 0.569 (0.197) | 0.857 |
| Platelet Crit Rate (%) | | 0.187 (0.068) | 0.183 (0.057) | 0.195 (0.089) | 0.278 |
| Urine pH (-) |  | 5.222 (0.420) | 5.198 (0.405) | 5.278 (0.452) | 0.244 |
| Serum Alanine Aminotransferase (U/L) | | 24.432 (22.662) | 24.603 (17.461) | 24.019 (32.169) | 0.874 |
| Serum Aspartate Aminotransferase (U/L) | | 25.984 (32.266) | 24.267 (13.193) | 30.148 (56.236) | 0.261 |
| Total Bilirubin (mmol/L) | | 15.551 (7.127) | 15.474 (6.965) | 15.737 (7.567) | 0.82 |
| Total Protein (g/L) | | 70.534 (6.328) | 69.962 (5.676) | 71.920 (7.566) | 0.055 |
| Albumin (g/L) | | 39.708 (3.740) | 39.999 (3.602) | 39.003 (4.002) | 0.1 |
| Globulin (g/L) |  | 30.848 (6.210) | 30.005 (5.164) | 32.893 (7.904) | 0.004 |
| Direct Bilirubin (mmol/L) | | 3.941 (2.329) | 3.721 (1.957) | 4.476 (3.005) | 0.045 |
| Serum Creatinine (mmol/L) | | 75.195 (67.362) | 75.069 (74.346) | 75.500 (46.858) | 0.969 |
| Blood Urea Nitrogen (mmol/L) | | 6.296 (2.457) | 5.978 (2.141) | 7.068 (2.977) | 0.006 |
| Uric Acid (mmol/L) | | 325.203 (102.293) | 328.111 (96.558) | 318.148 (115.703) | 0.548 |
| Fasting Blood Glucose (mmol/L) | | 6.508 (2.384) | 6.429 (2.148) | 6.699 (2.893) | 0.486 |
| Total Cholesterol (mmol/L) | | 4.548 (1.158) | 4.580 (1.154) | 4.471 (1.176) | 0.563 |
| Serum Low-Density Lipoprotein (mmol/L) | | 1.242 (0.520) | 1.203 (0.319) | 1.337 (0.822) | 0.111 |
| Serum High-Density Lipoprotein (mmol/L) | | 1.032 (0.178) | 1.038 (0.192) | 1.019 (0.136) | 0.495 |
| Triglycerides (mmol/L) | | 2.556 (0.903) | 2.473 (0.912) | 2.591 (0.900) | 0.423 |

Abbreviations: SD: Standard Deviation. SMI: Skeletal Muscle Index. BMI: Body Mass Index

Figure S1: Comparison of receiver operating characteristic curves among machine learning models in validation set


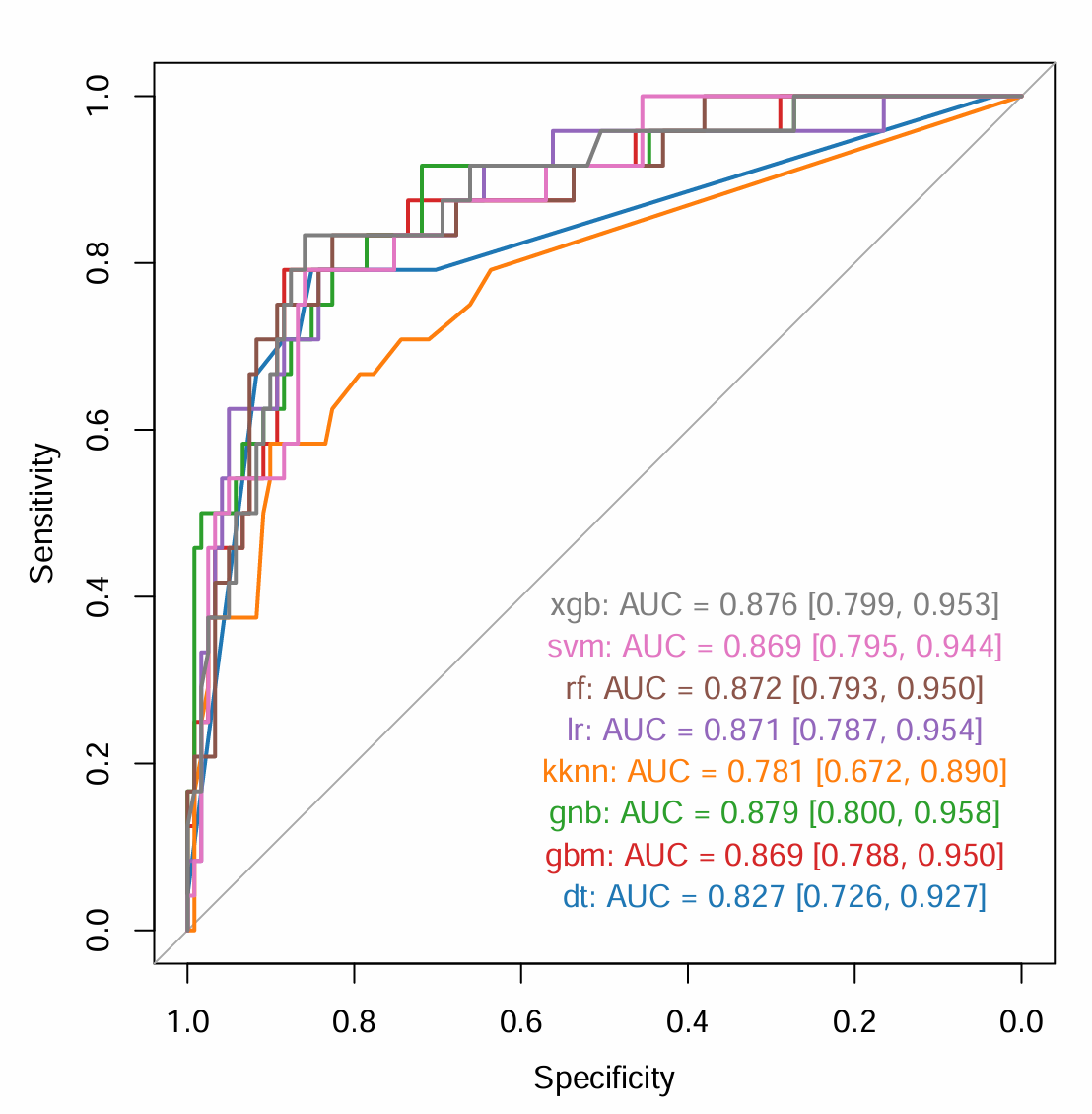


The plot compares model performance using sensitivity (y-axis) versus 1-specificity (x-axis). The diagonal dashed line represents random guessing (AUC=0.5).

Figure S2: Comparison of receiver operating characteristic curves among machine learning models in test set


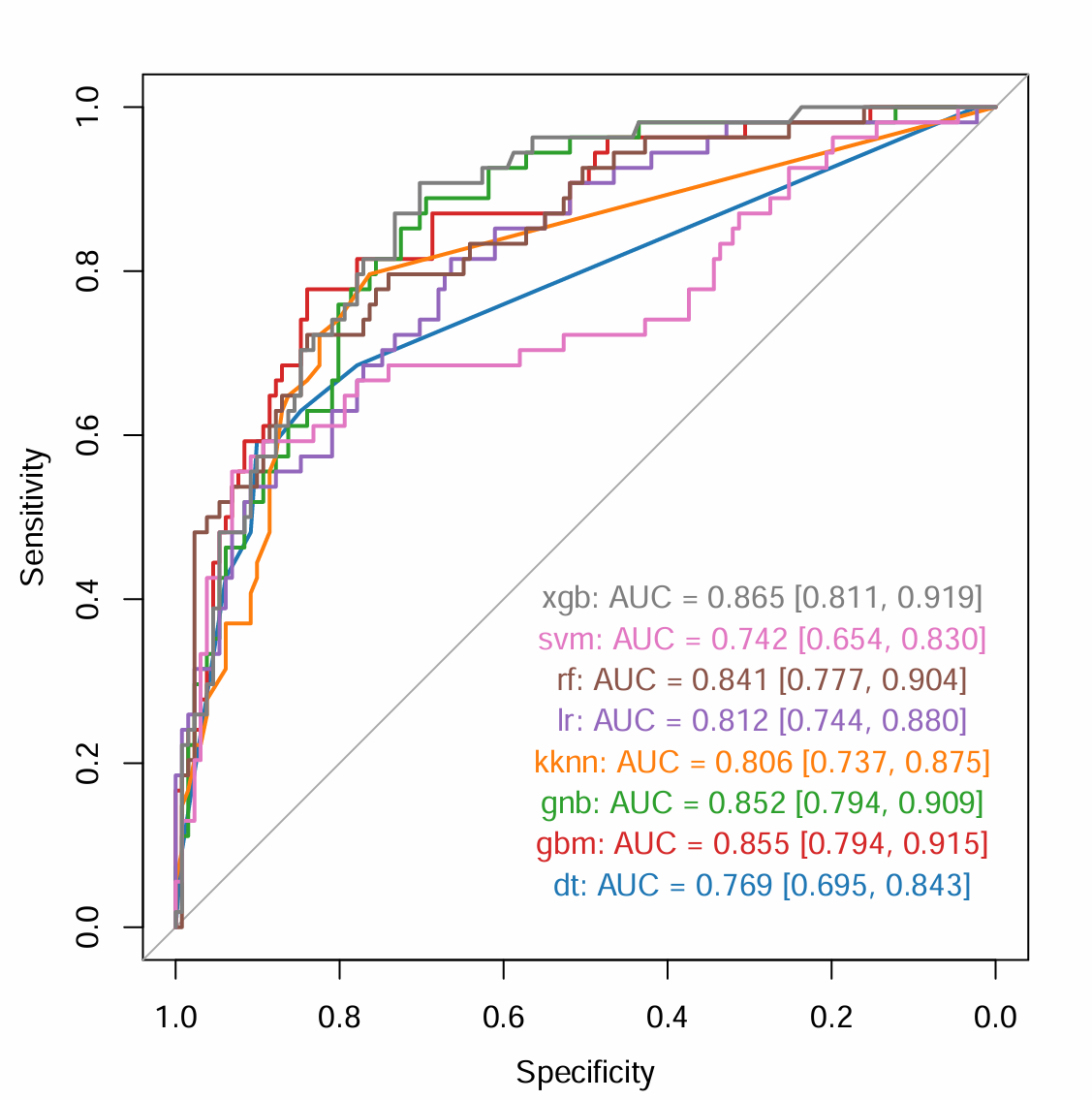


The plot compares prediction performance of eight models using sensitivity (y-axis) versus 1-specificity (x-axis). The diagonal dashed line represents random guessing (AUC=0.5).
